# Leisure Activities of Service Users in Psychiatric Rehabilitation– An Overlooked Avenue for Social Inclusion? Results from a Comparative Cross-Sectional Survey

**DOI:** 10.1007/s10597-025-01472-x

**Published:** 2025-06-17

**Authors:** Julia Häberli, Dirk Richter, Sonja Mötteli

**Affiliations:** 1 Centre for Psychiatric Rehabilitation, University Psychiatric Services Bern, Berne, Switzerland; 2 Department of Psychology, University of Bern, Berne, Switzerland; 3 University Hospital of Psychiatry and Psychotherapy, University of Bern, Berne, Switzerland; 4 Department of Health Professions, Bern University of Applied Sciences, Berne, Switzerland; 5 Department of Adult Psychiatry and Psychotherapy, University Hospital of Psychiatry Zurich, Zurich, Switzerland

**Keywords:** Leisure engagement, Leisure/recreational activities, Severe mental illness, Psychiatric rehabilitation, Social inclusion, Mental health

## Abstract

Leisure engagement plays a crucial role in people’s health. Although the effects of physical activity on mental health have been studied extensively, social leisure activities have been neglected so far. This study compares leisure satisfaction, behaviour and perceived barriers of 110 service users of sheltered workshops to those of 40 mental health professionals (MHPs). It examines factors associated with greater leisure satisfaction using a cross-sectional questionnaire. Main analyses were performed using ANCOVA and linear regression analyses. The frequency and variety of leisure activities were higher in MHPs (*p* < 0.001), with no difference in leisure satisfaction (but different perceived barriers) between the groups. Service users participating in regular group-based activities (42%) were more satisfied than service users who did not regularly participate (*p* < 0.05). Improving the service users’ leisure satisfaction might foster social inclusion and well-being. MHPs need to include those with the most severe mental health problems who usually refuse to participate in social activities.

## Introduction

Leisure engagement, defined as the amount of time spent in voluntary enjoyable activities outside of obligations such as work and domestic tasks, plays a crucial role in people’s physical and mental health. This connection is influenced by different mechanisms, including psychological, biological, social and behavioural processes (Fancourt et al., 2021). The relationship between leisure engagement and subjective well-being is mediated by leisure satisfaction with the frequency and variety of leisure activities appearing to be more important than the amount of time spent on an activity (Kuykendall et al., 2015).

Particularly for people with mental disorders, engaging in leisure activities can promote a more active lifestyle, reduce loneliness, isolation and boredom, regulate emotions and stress, structure time and thus have a positive impact on their recovery, life satisfaction and social inclusion (Fenton et al., 2017; Goodman et al., 2017; Iwasaki et al., 2010, 2014). The benefits of leisure experiences for people with mental disorders include social connections, psychological improvements, physiological benefits (including better mood and management of condition), physical health, practical skills, and cognitive improvements (Fenton et al., 2017). These benefits of leisure engagement as a therapeutic approach are increasingly recognised (Snethen et al., 2024). In addition to the well-established benefits of physical activity (Daumit et al., 2005; Noetel et al., 2024; Rosenbaum et al., 2014), studies are also highlighting the potential of other leisure activities, such as social activities and hobbies, as important adjunctions to conventional therapeutic approaches since they are associated with reduced depressive symptoms and lower negative affect (Bone et al., 2022; Dupuis & Smale, 1995; Fancourt & Steptoe, 2018; Goodman et al., 2016). There is evidence that regular group activities, such as participation in sports clubs or organized leisure activities, appear to be particularly beneficial for mental health (Street et al., 2007). However, not all leisure activities are good for health. For example, screen time-based sedentary behaviour, including watching TV, does not appear to generate psychological benefits (Kuykendall et al., 2015) and might even impair life satisfaction (Schmiedeberg & Schröder, 2017). Some leisure activities are also related to risks such as excessive use of media and harmful substances (Nimrod et al., 2012). In addition, people with very severe mental disorders often do not participate in any regular leisure activities (Høier et al., 2024). They often face internal and external barriers to participating in community leisure activities, such as illness-related lack of motivation, anxiety about social situations, no companionship, financial constraints and perceived stigma (Fenton et al., 2017).

Leisure engagement can contribute to the recovery and social inclusion of people with mental disorders. It might be a more malleable intervention than other life domains such as work and housing. Nevertheless, the potential of leisure engagement has largely been neglected by mental health professionals in the rehabilitation setting (Fenton et al., 2017; Iwasaki et al., 2014). Leisure behaviour of people with mental disorders, except for physical activity, is not yet well researched (Fenton et al., 2017; Rosenbaum et al., 2014). It also seems that people with mental disorders are not fully aware of the promising tool they could use as it has been shown that leisure satisfaction was equally high compared to the general population (Lloyd et al., 2001) with no desire for more activities in contrast to people with other impairments such as mobility disability (Páez & Farber, 2012).

Due to the reduced workload in rehabilitation facilities such as sheltered workshops, leisure time plays an important role in the lives of their service users. In line with the UN Convention on the Rights of Persons with Disabilities (United Nations, 2006), which demands a greater focus on the perspectives and preferences of those affected, a study was carried out to assess the potential need and interest of leisure support in people working in sheltered workshops (hereafter referred to as service users). For this purpose, we assessed their leisure satisfaction, behaviour, perceived restrictions and barriers to leisure engagement and interest in leisure support. We compared the service users’ leisure satisfaction, behaviour and perceived barriers to those of the mental health professionals working in the same rehabilitation institution (hereafter referred to as MHPs). Additionally, as proposed in previous research (Kuykendall et al., 2015), we examined whether group-based leisure activities are associated with greater leisure satisfaction in service users.

## Materials and Methods

### Setting

The sheltered workshops at the Centre for Psychiatric Rehabilitation of the University Psychiatric Services Bern (CPR UPD Bern) offer protected working environments addressed to people with severe mental health problems (mainly psychosis and affective disorders) who are deemed to be unable to work under the requirements of the general labour market. A total of 183 individuals (December 2023) worked in 11 different areas (e.g., gardening, sales, wood and textile workshop), mainly on production- and service-oriented activities with a reduced workload and lighter tasks supported by a supervisor. They received a limited salary for their work in addition to their disability pension. The sheltered workshops aim to enable participation in work life and social interaction by providing meaningful tasks and daily routines; however, they do not organize leisure activities.

### Participants and Procedure

**Fig. 1:**
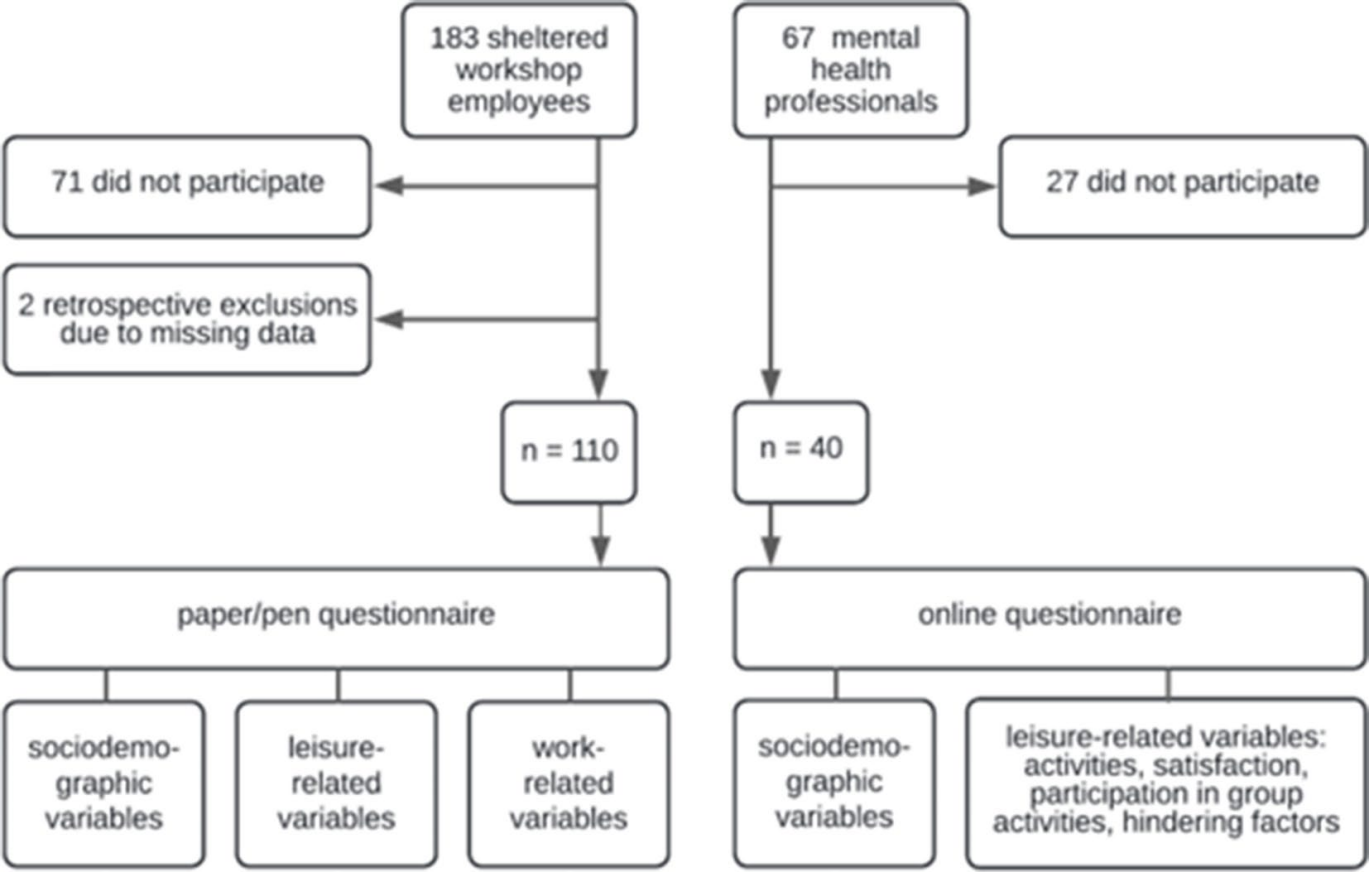
Flowchart

The study examines a research question that arose from the service users’ needs, and the questionnaire was developed in collaboration with MHPs working in sheltered workshops and a peer worker. After analysing the data, the findings were presented to and discussed with the service users (independently whether they participated in the survey or not) in one of two selectable sessions. The study followed a complete sampling, cross-sectional approach using a semi-quantitative questionnaire, meaning that all service users (*n* = 183) were invited to participate in the anonymous survey by their supervisors. In addition, all MHPs working in sheltered workshops (*n* = 35), day centres (*n* = 9) and supported employment (*n* = 23) at the CRP UPD Bern were also asked to participate (Fig. 1).

The questionnaire was developed using Qualtrics survey software. Two volunteers pretested the survey (online and paper-pencil versions). The service users were informed about the study’s aim and the voluntary and anonymous nature of the participation by their supervisors. Upon request of the service users, a paper-pencil version of the survey was administered over two months at the end of 2023. Questionnaires could be filled out during working hours or at home and had to be dropped anonymously into a letterbox. All participants verbally consented to participate in the anonymous questionnaire and data use for development and research purposes. A selection of supervisors was instructed to help fill out the surveys if needed. In total, 110 service users (response rate of 60.1%) filled out the questionnaire, which lasted about 20 min. In addition, 40 MHPs (response rate of 59.7%) participated in an online version (Fig. 1).

### Measures

#### Sociodemographic Variables

Gender (male, female, diverse), age (years), education and housing (answer options as depicted in Table 1) were assessed. Education (Matura and higher = 1, else = 0) and housing (living with family or friends = 1, else = 0) were dichotomized for analyses.

**Table 1:** Characteristics of *n* = 108 sheltered workshop users (service users) and *n* = 40 mental healthcare professionals (MHPs)

	Service users		MHPs	
	*n* or M	% or SD	*n* or M	% or SD
**Gender**				
male	60	55.6	15	37.5
female	48	44.4	25	62.5
**Age**	45.85	12.44	45.64	10.80
**Education**				
Not completed school education	7	6.5	0	0
Compulsory schooling	28	25.9	1	2.5
Vocational training	57	52.8	8	20.0
Matura (university entrance exam)	6	5.6	0	0
Higher vocational education	6	5.6	22	55.0
University	3	2.8	9	22.5
Unknown	1	0.9	0	0
**Housing situation**				
Living alone	43	39.8	11	27.5
Living with family or friends	30	27.8	29	72.5
Supported housing	8	7.4	0	0
Residential care home	16	14.8	0	0
Unknown	11	10.2	0	0
**Years of work in the institution**			-	-
< 1 year	21	19.4		
1–4 years	33	30.6		
5–10 years	22	20.4		
> 10 years	31	28.7		
Unknown	1	0.9		
**Workload (%)**	48.50	18.26	-	-
**Workdays per week**	3.87	0.93	-	-
**Reasons for work**			-	-
Daily routines	79	73.1		
Meaningful occupation	63	58.3		
Social exchange and support	52	48.2		
Participating in working life	50	46.3		
Learning new things	49	45.4		
Stability and resilience	41	38.0		
Improving self-confidence	41	38.0		
Not being at home too much	36	33.3		
Improving health	29	26.9		
Preparing for general labour market	12	11.1		

*Notes.* Missing values in age (service users: *n* = 12, MHPs: *n* = 1), workload (*n* = 1) and workdays per week (*n* = 1)

#### Leisure-related Questions

Participants were asked to rate their overall satisfaction with their leisure activities on a scale ranging from ‘0 = not satisfied at all’ to ‘10 = very satisfied’. Questions related to leisure activities were assessed using both a subjective and structural approach (Kuykendall et al., 2015; Newman et al., 2014). In the subjective approach, participants were asked to name activities undertaken in their leisure time (open question). In the structural approach, the frequency with which 16 predefined types of leisure activities were carried out (as depicted in Table 2) was assessed with the answer options “4 = daily”, “3 = several times a week”, “2 = several times a month”, “1 = several times a year” and “0 = never”. The categories were explicitly developed for the study’s purpose and guided by previous research (Peterson et al., 2020; Takiguchi et al., 2023). We only reported the quantitatively assessed leisure activities; the qualitative ones added no additional information. Mean activity was calculated based on the frequency of all categories. In addition, a variety score of leisure activities was calculated as the sum of individual activities (never = 0, all other categories = 1). The factors that hinder practising leisure activities were also assessed by the open question “Are there any leisure activities that you would like to do but can’t? Which ones and why?».

**Table 2:** IMET scores - restrictions in participation in different dimensions of life of sheltered workshop users (service users) compared to norm values

	Male service users				Female service users			
Item	M	SD	*N*	Z-score	M	SD	*N*	Z-score
Activities of daily living (e.g., washing, eating)	2.05	2.05	60	1.52	2.23	2.19	47	1.72
Activities at home (e.g., housework, gardening)	2.53	2.01	58	1.87	2.87	2.15	47	2.22
Activities outside the home (e.g., shopping, driving around, doctor visits)	3.03	2.70	60	2.46	2.89	2.50	47	2.33
Duties (e.g., cleaning up, care of others, work, school)	2.66	2.49	59	2.01	2.38	1.72	45	1.74
Recreational activities (e.g., sports, hobbies, leisure time)	2.32	2.06	59	1.56	3.17	2.58	47	2.45
Social activities (e.g., meeting friends, eating out, going to the theatre)	3.75	3.38	60	3.09	3.28	2.86	47	2.65
Close relations (e.g., partner, family)	4.33	3.14	60	3.71	2.85	2.65	46	2.26
Sexual life*	-	-	-	-	-	-	-	-
Coping with stress and extraordinary strain (e.g., conflicts with family, stress at work)	4.59	2.95	59	3.63	5.04	2.90	46	4.04
**Mean IMET scores**	**3.12**	**1.91**	**60**	**2.41**	**3.13**	**1.81**	**48**	**2.44**

Notes. Higher values indicate higher restrictions in participation; *not assessed; norm values refer to a population-based survey in Northern Germany (Deck et al., 2015)

Regular participation in group activities was assessed by the question “Do you regularly participate in group activities (e.g., sports club, recreational courses, meeting with friends, etc.)?”. The answer format was yes or no.

Also, illness-related restrictions in participation in different dimensions of life, including leisure time, were assessed using the validated IMET scale (index for the assessment of health impairments) ranging from ‘0 = no impairment’ to ‘10 = no activity possible any more’ (Deck et al., 2011, 2015). We compared the IMET scores with published norm values of a population-based survey in Northern Germany (Deck et al., 2015) using calculated z-scores.

Further, participants were asked whether they would be interested in leisure activities organized by their rehabilitation institution: “How interested are you in leisure activities organised by your institution (e.g., joint walks, yoga, fitness, tennis, dance, museum/theatre/concert visits, mini-golf, games afternoons, literature club, cooking classes, etc.)? The answer options were “not interested”, “less interested”, “rather interested”, and “very interested”.

#### Work-related Questions

Participants were asked about their working field, workload (percentage and days per week), years of work in the sheltered workshop, the reason for working in the sheltered workshop (multiple choice options as depicted in Table 1), the overall satisfaction with their workplace on a scale ranging from ‘0 = not satisfied at all’ to ‘10 = very satisfied’ and whether they would be interested in getting a paid job in the primary labour market (yes, yes but only in the future, no).

### Analyses

Descriptive analyses were based on means (M) and standard deviations (SD) for continuous and frequencies for categorical variables. Scale scores, such as the mean of IMET, were calculated if at least 66% of the items were completed. We used mean imputation for single missing values in age (8.8%), satisfaction with leisure activities (1.4%) and IMET (2.8%). We used zero imputation for categorical leisure activities (15%) as, based on the agreement of qualitative and quantitative data, missing activities likely indicate irrelevant activities. Differences in socio-demographic variables were tested using Chi-Square tests and T-tests for independent samples. An ANOVA was calculated to examine the relationship between perceived restrictions (IMET) and interest in leisure support in service users. Mean differences in leisure satisfaction, mean activity, and variety scores between service users and MHPs were tested using ANCOVA, including the control factors of gender, age, education, and living situation. In addition, a linear regression analysis was conducted to examine the effect of regular participation in group activities on leisure satisfaction in service users including the control factors of gender, age, education, living situation, and IMET. Unstandardized regression coefficient (B), standard errors (SE) and effect size (f2) for the predictor group-participation were calculated. The statistical analyses were performed with the software R (version 4.3.0; R Core Team, 2024). For all tests, the significance level was set at 5%.

## Results

### Participant Characteristics, Working Satisfaction and Goals of Service Users

Data from 108 service users and 40 MHPs were analysed; data from two service users had to be excluded because of only missing values in sociodemographic and leisure variables. Service users’ ages ranged between 20 and 68 years, with a higher proportion of males and a majority having vocational education. Half of them worked in the sheltered workshops for over 5 years, with an average workload of 50% (range 10 to 100%). The average workplace satisfaction was high (M = 8.43, SD = 1.78), with 30% interested in a paid job in the general labour market (8.3% yes, 21.3% yes, but only in the future). MHPs’ age was, on average, 45.64 years (SD = 10.66), and the comparison sample consisted of 63% females (Table 1). Age and gender did not significantly differ between the service users and MHPs (*p* = 0.921, *p* = 0.077), whereas education and housing situation significantly differed between the groups (*p* < 0.001 in both cases).

### Perceived Restrictions and Support Needs for Leisure Engagement in Service Users

The users’ overall IMET score, encompassing restrictions in participation in different dimensions of life, was M = 3.12 (SD = 1.86) and was significantly higher than the norm values (Table 2). Thereby, a higher score was mainly due to perceived restrictions related to coping with stress and extraordinary strain, close relations, and social activities such as meeting friends and eating out than restrictions related to recreational activities such as sports and hobbies (Table 2). Users with higher IMET scores, meaning more perceived restrictions, were significantly less likely interested in participating in institution-organized leisure activities (F = 9.61, *p* = 0.003, *n* = 106): of all participants, 23% were not interested (M_IMET_ = 3.70, SD_IMET_ = 2.03), 31% were less interested (M_IMET_ = 3.43, SD_IMET_ = 1.85), 28% were rather interested (M_IMET_ = 3.18, SD_IMET_ = 1.44) and 17% were very interested (M_IMET_ = 1.91, SD_IMET_ = 1.77).

### Leisure Satisfaction, Perceived Barriers and Activities in Service Users and MHPs

Overall, 64% of the service users and 60% of the MHPs were very satisfied with their leisure engagement (8–10 points) with no significant group effect on leisure satisfaction (M = 7.52, SD = 2.22 vs. M = 7.80, SD = 1.57, F (1,142) = 0.55, *p* = 0.459) while controlling for the covariates of gender, age, education, and living situation.

In total, 31% of the service users and 58% of the MHPs perceived barriers that hinder them from practising desired leisure activities, with different reasons (service users: 12% health, 11% financial constraints, 2% not enough time, 6% other reasons vs. MHPs: 5% health, 13% financial constraints, 40% not enough time, 0% other reasons).

Mean activity was significantly lower in service users than MHPs (M = 1.57, SD = 0.51 vs. M = 1.88, SD = 0.34, F (1,142) = 13.60, *p* < 0.001) while controlling for the covariates. Similarly, the variety score, reflecting the sum of a person’s different leisure activities, was also significantly lower in the service user group than in MHPs (M = 10.91, SD = 3.27 vs. M = 13.97, SD = 1.49, F(1,142) = 34.94, *p* < 0.001) while controlling for the covariates.

**Fig. 2:**
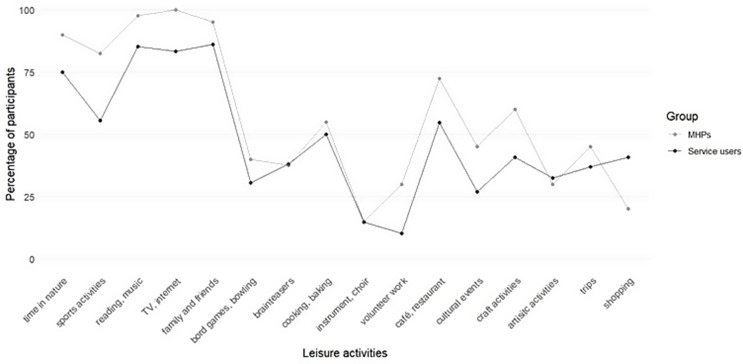
Percentage of participants who perform leisure activities at least several times a month

Table 3; Fig. 2 show detailed differences in the frequency of leisure activities between the service users and MHPs, which were mainly higher in MHPs. In addition to the 16 different categories, 15 service users and 9 MHPs mentioned additional activities such as spending time with/caring for animals and relaxing/doing nothing.

**Table 3:** Differences in leisure activities between *n* = 108 sheltered workshop users (users) and *n* = 40 mental healthcare professionals (MHPs)

Leisure activities	several times a week to daily (%)		several times a month (%)		never to several times a year (%)	
	Users	MHPs	Users	MHPs	Users	MHPs
Take a walk, spend time in nature	58	56	17	35	25	10
Sports activities	32	58	23	25	45	18
Reading, listening to music	74	88	11	10	15	3
TV, internet, social media, computer games	80	95	4	5	17	0
Time with family and friends	51	78	35	18	14	5
(Board)games, bowling	8	11	22	30	69	61
Brain teasers, foreign languages	19	26	19	13	62	63
Cooking and baking for guests	24	13	26	43	50	45
Musical instrument, choir, band	7	8	8	8	85	85
Volunteer work	4	16	6	15	90	71
Café, restaurant, bar	24	13	30	60	46	28
Cultural and sports events	6	0	21	45	73	55
Craft activities, gardening, knitting	20	30	21	30	59	40
Artistic activities such as painting or photography	19	15	14	15	67	70
Excursions/trips by bus, train, boat or car	15	3	22	43	63	56
Shopping (not for household)	16	3	24	18	60	81

*Notes*. For better legibility, percentages were rounded to the nearest whole number resulting in +/-100%

### Regular Participation in Group Activities Predicts Leisure Satisfaction in Service Users

In total, 42% of the service users, in comparison to 75% of the MHPs, regularly participate in group activities. Thereby, gender and age were equally distributed. A multiple linear regression analysis was conducted to examine the effect of regular group participation on leisure satisfaction in service users while controlling for gender, age, education, living situation and IMET scores. The whole model explained 38% of the variance in leisure satisfaction (adjusted R^2^ = 0.35, F (6,101) = 10.5, *p* < 0.001). Gender (B = -0.76, SE = 0.35, t = -2.13 *p* < 0.05) and IMET scores (B = -0.52, SE = 0.09, t = -5.23, *p* < 0.05) were significant predictors besides regular participation in group activities (B = 1.26, SE = 0.37, t = 3.38, *p* = 0.001, f^2^ = 0.11).

## Discussion

In this study, we wanted to examine whether and what kind of support people with long-lasting mental health problems would like to receive in the leisure sector, regarding the overarching aim of improving social inclusion and well-being in the rehabilitation setting. For this purpose, leisure satisfaction, behaviour, perceived barriers to leisure engagement and interest in participating in organized activities were examined in a group of sheltered workshop users and compared to a healthy control group of MHPs working in the same rehabilitation institution.

The results show that service users perceived higher restrictions to participation in social (leisure) activities than the general population, had lower activity levels and participated less often in regular group-based activities. Thereby, the interest in organized leisure support was lowest among those with the highest impairment scores. This might pose a challenge for practical implications as participation in social leisure activities has been associated with better mental health (Bone et al., 2022).

The service users had an average workload of 50%, meaning that leisure takes up much of their time. One of the service users’ main goals in working in sheltered workshops is to create daily routines. Similarly, but even more malleable, leisure engagement can also contribute to establishing daily routines, particularly for those with lower workloads or unemployment (Goodman et al., 2017). Besides that, it can promote a more active lifestyle and improve social inclusion (Fenton et al., 2017). Therefore, it could be seen as a missed chance in rehabilitation settings not to take advantage of the opportunities that leisure engagement brings.

Consistent with previous findings (Deck et al., 2011, 2015), the service users perceived illness-related restrictions in participation in different dimensions of life, particularly in social-related (leisure) activities, indicating a need for leisure support. However, service users with the highest impairment scores (IMET) were less interested in participating in leisure activities organised by their institution than service users with lower impairment scores.

In line with previous findings (Fenton et al., 2017; Rosenbaum et al., 2014), the frequency and variety of leisure activities performed were higher in MHPs for most activities. Also, most MHPs but less than half of the service users participated in regular group activities. However, the service users who regularly participated in group-based activities were significantly more satisfied with their leisure engagement regardless of their IMET scores. This result aligns with previous findings showing that group-based (social) leisure activities seem particularly beneficial for mental health (Street et al., 2007). Because people with mental health problems are often less socially involved and engage in fewer leisure activities than the average population due to many perceived barriers, such as lack of companions, motivation or finances (Fenton et al., 2017), one might assume that they should be less satisfied with their leisure engagement. However, leisure satisfaction was equally high among service users and MHPs, which was also found in a previous study despite the authors’ contradictory assumptions (Lloyd et al., 2001). The results of this study add to the literature by providing a possible explanation for this finding. The MPHs perceived nearly twice as many barriers to leisure engagement as the service users (58% vs. 31%). The main reason for MPHs was time constraints, which is understandable given the high average workload. Consistent with previous findings (Fenton et al., 2017), service users indicated barriers to leisure engagement, such as poor health and financial constraints. However, the low percentage of service users who reported barriers contradicts the average IMET scores for social and leisure activities. It seems that people with mental health problems, perhaps due to their mental illness, have lower personal expectations of their leisure time than the average population. In addition, socially isolated people may not even know their possibilities. Another reason may be the institutional setting of the survey: service users, as service recipients, may have been less critical, whereas MPHs, as service providers, may have been more critical.

### Practical Implications

Our results show a quandary between the potential use and benefit of institution-organized leisure support for sheltered workshop users, which causes a challenge for practical implementation: participating in regular group or social activities seems beneficial for leisure satisfaction and, thus, social inclusion and well-being. However, service users with higher impairments and more need for support indicated less interest in participating in institution-organized leisure activities. Nimrod et al. (2012) described a vicious circle regarding coping with leisure and depression. People with depression perceive leisure as a useful coping resource but are trapped in this circle since the more depressed they feel, the less they can participate in leisure activities and vice versa. Their study strongly supports the dimensionality of leisure concerning depression as both part of the problem and the solution. This vicious circle should not be accepted as such, but there should be a search for possible interventions to break it. Our findings suggest that common (unspecific) group activities to improve social inclusion and well-being offered by psychiatric rehabilitation facilities, as recommended in the literature (Fenton et al., 2017), seem to be aimed at the wrong target group. Less impaired service users are interested in institutionalized activities but may be better supported in the community setting for rehabilitation and social inclusion purposes. In contrast, more impaired service users may benefit greatly from participation in protected group activities but would not participate voluntarily. Therefore, more research is needed on how to specifically target and motivate those with the most severe mental health problems, as this group is otherwise difficult to reach.

### Limitations

Although this study emphasised important aspects of leisure activities for people working in sheltered workshops, there are several limitations to consider. First, the data was only collected in one institution, meaning the results cannot be generalized to other settings. Second, the data was collected using a cross-sectional questionnaire, which does not allow for causal conclusions. Third, although this reflects reality, education and housing situations differed strongly between the service users and the MHPs. For both questionnaires, the assessed leisure activities seemed to cover the participants’ leisure behaviour well, but it needs to be supplemented in further studies with the categories ‘spending time with pets’ and ‘relaxing’.

## Conclusion

Supporting leisure engagement in people with mental health problems might be a promising, but still underestimated, way to improve social inclusion and well-being in psychiatric rehabilitation, which has focused more on occupational and housing-related support so far. Apart from the support of individual preferences, regular group-based activities are potentially beneficial for improving people’s leisure satisfaction. Mental health professionals need to develop strategies to motivate and include those with the most severe mental health problems who usually refuse to participate in social activities.

## Data Availability

Due to data sensitivity (belonging to a psychiatric institution), data cannot be shared publicly. If you are interested, please contact the corresponding author.
